# From Structure to Luminescent Properties of B_2_O_3_-Bi_2_O_3_-SrF_2_ Glass and Glass-Ceramics Doped with Eu^3+^ Ions

**DOI:** 10.3390/ma14164490

**Published:** 2021-08-10

**Authors:** Karolina Milewska, Michał Maciejewski, Anna Synak, Marcin Łapiński, Aleksandra Mielewczyk-Gryń, Wojciech Sadowski, Barbara Kościelska

**Affiliations:** 1 Faculty of Applied Physics and Mathematics, Institute of Nanotechnology and Materials Engineering, Gdańsk University of Technology, ul. Gabriela Narutowicza 11/12, 80-233 Gdańsk, Poland; michal.maciejewski@pg.edu.pl (M.M.); marcin.lapinski@pg.edu.pl (M.Ł.); alegryn@pg.edu.pl (A.M.-G.); wojciech.sadowski@pg.edu.pl (W.S.); 2 Faculty of Mathematics, Physics and Informatics, Institute of Experimental Physics, University of Gdańsk, ul. Wita Stwosza 57/246, 80-952 Gdańsk, Poland; anna.synak@ug.edu.pl

**Keywords:** glass, glass-ceramics, luminescence, nanocrystals

## Abstract

Glass-ceramics with the composition B_2_O_3_-Bi_2_O_3_-SrF_2_ were synthesized by the conventional melt-quenching technique and subsequent crystallization of the parental glasses. The temperature at which the ceramization was carried out was selected based on differential scanning calorimetry (DSC) analysis. The structure of the studied materials and the formation of SrF_2_ nanocrystals were confirmed by the Fourier-transform infrared spectroscopy (FTIR), X-ray diffraction (XRD), and X-ray photoelectron spectroscopy (XPS) techniques. It was found that the amount of strontium fluoride introduced into the parental borate-bismuth glass has a significant impact on the growth of SrF_2_ nanocrystals. In particular, the influence of the crystalline SrF_2_ phase on luminescence intensity and kinetics was studied using Eu_2_O_3_-doped samples. An increase in luminescence intensity was observed in the samples in which SrF_2_ nanocrystals were formed. This is most likely related to the fact that some of the Eu^3+^ ions were (after annealing of the glass) located in the crystalline structure of strontium fluoride. This was confirmed both by the luminescence lifetime obtained based on the luminescence decay curves and the calculated Judd–Ofelt parameters, Ω_2_ and Ω_4_. The results achieved confirm that the glasses and glass-ceramics described in this work could be considered as a new phosphor for light-emitting diodes (LEDs).

## 1. Introduction

Glass as a material for applications in optics and optoelectronics has enjoyed unflagging interest for many years. In particular, a lot of research is related to glasses and glass-ceramics with optically active dopants such as rare-earth ions, which are promising materials for use in white LEDs [1,2,3]. This is because of their unique combination of properties for excellent transmission in the visible and infrared spectral range, high refractive index, and good chemical and mechanical stability [4,5]. Glasses that are the matrix for the rare-earth ions should also have low phonon energy, which decreases the risk of multi-phonon relaxation processes and, consequently, of non-radiative transitions [6]. When we consider glasses for these applications, borate glasses seem to have great potential. Boron oxide as a glass former is characterized by a wide glass formation range, high transparency, and high thermal stability [7]. Unfortunately, the addition of some network modifiers to boron oxide, usually alkali oxides, can lead to a change of coordination number of some of the boron atoms from 3 to 4, changing the properties of borate glass. Interestingly, the property change does not occur linearly with the amount of modifier. This phenomenon is known as the borate anomaly [8,9,10]. The modifier that allows very good optical properties of borate glasses to be obtained is bismuth oxide. Borate-bismuth glasses are characterized by high density and high refractive indices and, importantly due to their preparation, are glasses with a comparatively low melting point in a wide glass formation range (20–80 mol % Bi_2_O_3_) [11]. The choice of bismuth oxide over lead oxide, which has often been used in glasses in recent years, seems particularly good, as it breaks down the toxic material while leaving the glass with the desired properties. However, the addition of Bi_2_O_3_ to borate oxide also changes the coordination number of the boron atoms [10,12,13], changing the properties of the resulting glass.

Borate glasses were often used as matrices for RE^3+^ ions [12,14,15]. However, research devoted to this subject has shown that an interesting alternative to glasses are glass-ceramics, especially those containing metal fluoride nanocrystals. Optical transparency of glass-ceramics can be reached if the crystallite diameter does not exceed 30 nm [16]. Crystal lattice, especially when the crystalline phase is fluoride, prevents them from luminescence concentration quenching due to clusters of RE^3+^ ions forming [17,18,19]. Furthermore, if there are heavy atoms in the structure of the nanocrystals, an additional factor appears in the amorphous matrix that reduces the phonon energy of the matrix what consequently promotes RE^3+^ radiative transitions, keeping all the benefits of the glass matrix at the same time [17,20,21,22,23]. Much research has been related to the existence of PbF_2_ nanocrystals in the borate matrix [14,15]. However, as mentioned earlier, lead is being phased out due to its toxicity. Among various fluoride nanocrystals, which are more environmentally friendly than lead compounds, strontium fluoride SrF_2_ seems to be a very good choice in terms of materials for optical applications. This is because SrF_2_ exhibits a wide bandgap, low phonon energy, and relatively low hygroscopic properties [24]. On the other hand, to the best of our knowledge, there is no information in the literature on the possibility of crystallization of SrF_2_ in a borate-bismuth matrix. SrF_2_ nanocrystals are usually grown in glasses by annealing them at a suitable temperature above the glass transition temperature (T_g_). The size of nanocrystals can be then controlled by changing heat treatment parameters, but it also depends on the type of glass structure [23,24,25,26,27]. In the case of borate-bismuth glass, the problem is more complicated because in this glass it is possible to obtain at least five stable crystalline phases of Bi_2_O_3_-B_2_O_3_ [11] and metastable bismuth orthoborate phases [28]. They can also crystallize in the matrix during annealing, leading to the crystallization of the SrF_2_ phase. Nevertheless, due to the optical properties of borane-bismuth glasses and the known beneficial effect of SrF_2_ nanocrystals on the luminescence of rare-earth ions, it is worth undertaking such research.

This work is devoted to the synthesis of borate-bismuth glasses and glass-ceramics containing SrF_2_ nanocrystals. We present here the results of structural studies of the above materials, as well as the impact of annealing the glasses on the luminescent properties of Eu^3+^ ions incorporated in them. The research was carried out to assess these materials as potential candidates for LED phosphors.

## 2. Materials and Methods

Borate-bismuth glasses with a nominal composition (in mol%) of 50B_2_O_3_-50Bi_2_O_3_ (BBO), 45B_2_O_3_-45Bi_2_O_3_-10SrF_2_ (BBO+10SrF_2_), and 40B_2_O_3_-40Bi_2_O_3_-20SrF_2_ (BBO+20SrF_2_) were synthesized using the conventional melt quenching technique. Moreover, glasses doped with Eu_2_O_3_ (2 mol %) were prepared: BBO+Eu, BBO+10SrF_2_+Eu, and BBO+20SrF_2_+Eu. Well-mixed starting raw materials H_3_BO_3_, Bi_5_OH(OH)_9_(NO_3_)_4_, SrF_2_, and Eu(NO_3_)_3_ were melted in a porcelain crucible at 1100 °C for 15 min. After that, melts were poured onto a steel hot plate (~250 °C) and immediately pressed by another plate, and then cooled down to room temperature. To investigate the effect of annealing the glasses on the crystallization of SrF_2_ nanocrystals, the samples were annealed in the interval of 450–590 °C for 1 h and 24 h in an air atmosphere. 

Thermal properties of as-prepared glasses were studied on a Netzsch Simultaneous Thermal Analyzer, STA 449 F1, in the platinum-rhodium crucible in an air atmosphere, with a heating rate of 10 K/min. Before each measurement, a blank calibration run was conducted to account for the buoyancy effect. Differential scanning calorimetry (DSC) allowed the characteristic temperatures to be determined, such as the glass transition temperature Tg and crystallization temperature Tc.

The amorphous nature of the glasses as well as the presence of crystalline phases present in them as a result of annealing was confirmed in X-ray diffraction (XRD) studies. XRD measurements were performed on powder samples on a Philips X’PERT PLUS diffractometer with Cu-Kα radiation.

To determinate the types of structural units present in the samples, Fourier transform infrared spectroscopy (FTIR) measurements were carried out. The measurements were performed on a Perkin-Elmer Frontier MIR/FIR spectrometer with a TGS detector on pellet samples mixed with potassium bromide KBr in a weight ratio (Sample:KBr) of 1:100. 

X-ray photoelectron spectroscopy (XPS) analysis confirming the valence states of ions present in the samples was carried out with an Omnicron NanoTechnology spectrometer with a 128-channel collector. XPS measurements were performed in ultra-high vacuum conditions, below 1.1 × 10^−8^ mBar. The photoelectrons were excited by an Mg-Kα X-ray source with X-ray anode operated at 15 keV and 300 W. 

Luminescence emission and excitation spectra of the samples were collected by a SCINCO FluoroMate FS-2 fluorescence spectrometer using pellet samples mixed with KBr in a weight ratio of 1:1. The single measurement results were obtained as a quasi-three-dimensional, colorful flat image, with the wavelength in the horizontal axis, the time in the vertical axis, and the intensity expressed by a range of colors.

Time-resolved emission spectra (TRES) were acquired using a pulsed spectrofluorometer described in detail [27]. The laser system PL2251-20 with an Nd:YAG laser and a PG 401/SH optical parametric generator emitting pulses of FWHMz30 ps from EXSPLA was used as the excitation light source. The emission signal was analyzed by a Bruker Optics 2501S spectrograph and the Hammamatsu streak camera C4334-01 model. All operations were fully automated and controlled by the original Hamamatsu HPDTA software, which allows for real-time data analysis. By slicing the streak camera image at a certain time interval, the time decays were obtained.

## 3. Results and Discussion

### 3.1. DSC Analysis

**Figure 1:**
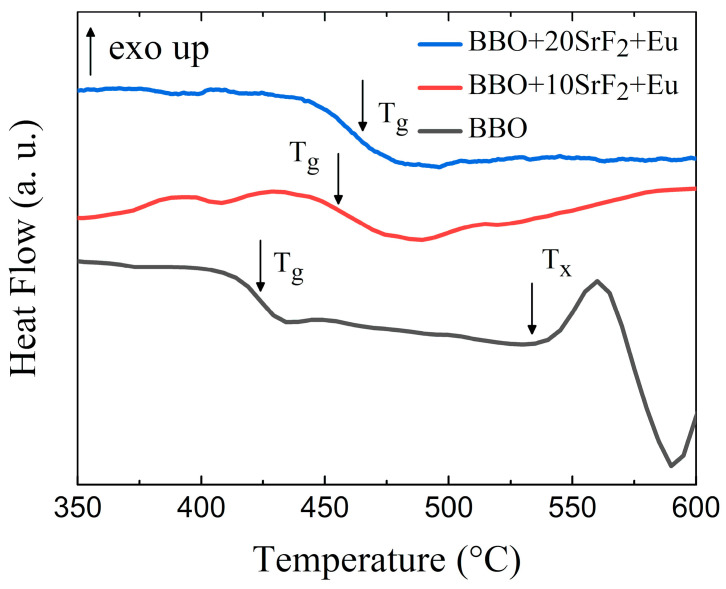
DSC curves of BBO, BBO+10SrF_2_, and BBO+20SrF_2_ glasses.

To analyze the thermal properties and the thermal stability of the prepared glasses, DSC measurements were performed. The results are presented in Figure 1. One can observe that the glass transition temperature depended on the SrF_2_ content. T_g_ was located around 424 °C, 456 °C, and 459 °C for BBO, BBO+10SrF_2_, and BBO+20SrF_2_ glasses, respectively. Interestingly, the exothermic maximum associated with crystallization was only visible for the BBO sample. The onset of crystallization peak temperature (T_x_) was located at 534 °C, which is well below the crystallization temperatures of the possible crystalline phases [11]. The glass stability region ∆T, defined as the difference between T_x_ and T_g_, for BBO glass was equal to 110 °C. This is a wide range, but even a small quantity of B_2_O_3_ may have a positive effect on the thermal stability of this glass [29]. Taking into account the crystallization of the glasses, the thermal stability S parameter describing the glass resistance against devitrification was also determined [30]:

(1) $$S=\frac{(T_{c}-{\;T}_{x})\left(T_{c}-T_{g}\right)}{T_{g}}$$

In studied BBO glass, the calculated value of S is 8.72. For comparison, the values of S found in the literature for other borate glasses was even about 1 (50B_2_O_3_-50Bi_2_O_3_ [31], 50Li_2_O-50B_2_O [32]). Unfortunately, such a significant difference between these values and the value obtained for our glass was not clear to us. As can be seen, the addition of SrF_2_ to the base BBO composition led to the disappearance of the crystallization peak. Therefore, it can be said that it prevented the crystallization of the matrix. Unfortunately, no effect related to the crystallization of the SrF_2_ phase in the BBO matrix was observed in these glasses. 

### 3.2. XRD Analysis

**Figure 2:**
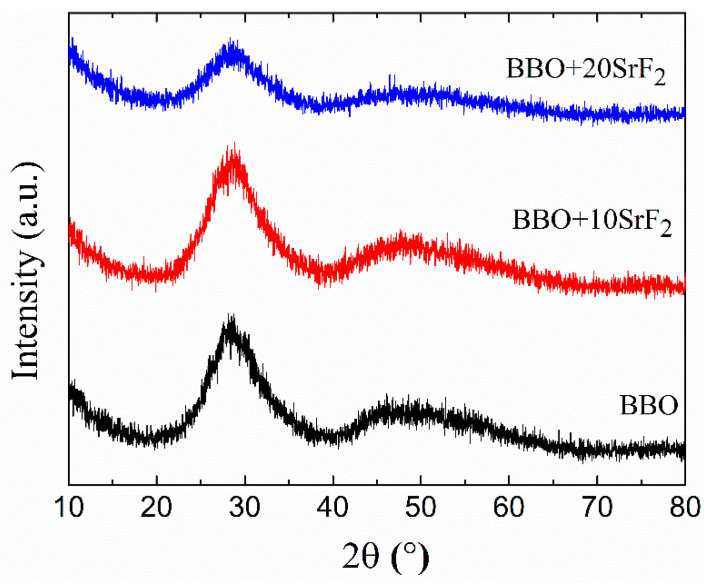
XRD patterns of as-prepared BBO, BBO+10SrF_2_, and BBO+20SrF_2_ glasses.

The amorphous character of as-prepared glasses was confirmed by XRD studies. Figure 2 shows the diffraction patterns of BBO, BBO+10SrF_2_, and BBO+20SrF_2_ glass samples. Only broad halos associated with amorphous materials can be seen.

**Figure 3:**
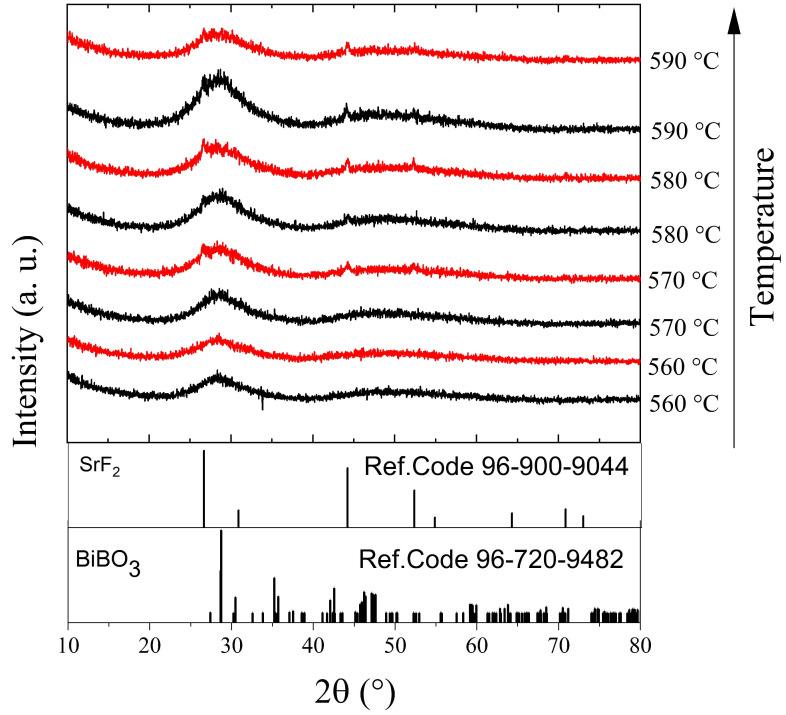
XRD patterns of BBO+10SrF_2_ (black) and BBO+20SrF_2_ (red) glasses after annealing in the temperature range of 560–590 °C for 1 h.

**Figure 4:**
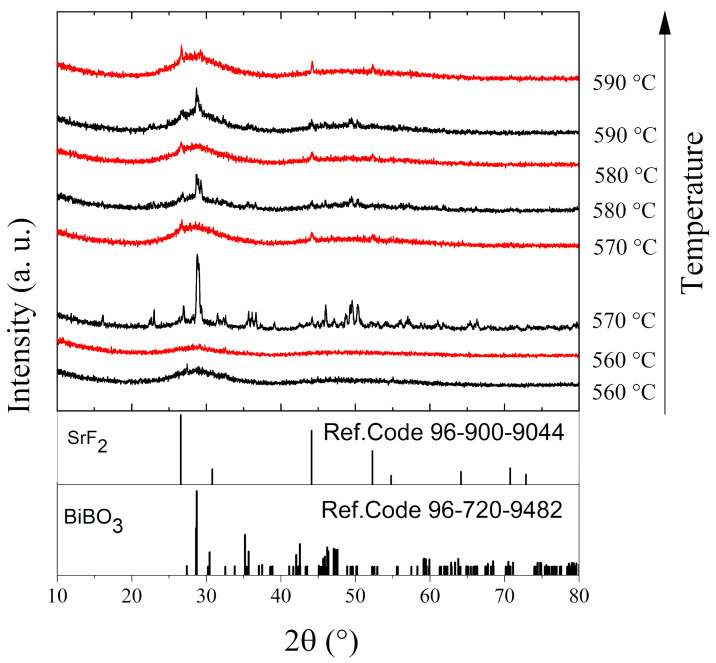
XRD patterns of BBO+10SrF_2_ (black) and BBO+20SrF_2_ (red) glasses after annealing in the temperature range of 560–590 °C for 24 h.

The effect of the annealing temperature on the structure of the BBO+10SrF_2_ and BBO+20SrF_2_ glasses is shown in Figure 3 and Figure 4. In Figure 3, X-ray diffractograms for the BBO+10SrF_2_ and BBO+20SrF_2_ glasses annealed at temperatures ranging from 560 to 590 °C for 1 h are presented. Figure 4 presents XRD patterns of these glasses after heat treatment in the same temperature range, but for 24 h. The aim of the thermal treatment was the growth of SrF_2_ nanocrystals in the glass matrix. XRD patterns of the samples annealed at 560 °C did not differ from the patterns of the unannealed samples and showed a lack of long-range order. It should be noted, however, that in the presented XRD results a small amount of the nano-sized crystalline phase could have gone unnoticed, especially since the amorphous phase in which the nanocrystals were dispersed gives a broad halo in the range of 25–35 (2θ), in which there may be major reflections derived from the crystalline phases. Clear differences between BBO+10SrF_2_ and BBO+20SrF_2_ samples appeared after annealing at 570 °C. XRD patterns of the BBO+10SrF_2_ sample showed crystallization mainly in the BiBO_3_ phase (Ref.Code 96-720-9482), but there were also visible low-intensity reflections corresponding to the SrF_2_ phase (Ref.Code 96-900-9044). Unfortunately, there were also unidentified peaks in the diffractograms that were likely related to bismuth oxides, but crystalline borates sometimes have highly unusual stoichiometries [8]; therefore, it is difficult to fully characterize the presented diffraction pattern. On the other hand, BBO+20SrF_2_ samples showed only the peaks characteristic of the SrF_2_ crystalline phase (Ref.Code 96-900-9044). As can be seen, with the increase in the annealing temperature, the amount of BiBO_3_ crystalline phase in the BBO+10SrF_2_ samples decreased, whereas a weak reflection of the SrF2 phase was already present in the sample annealed at 580 °C. In the case of the annealed BBO+20SrF_2_ glass, the SrF_2_ phase crystallized regardless of the temperature increase. The obtained diffractograms confirm the conclusion drawn based on DSC studies that the presence of SrF_2_ in the BBO matrix prevented its crystallization. Unfortunately, annealing the samples at temperatures of 570 °C and higher caused them to begin to lose their transparency. Therefore, 560 °C was chosen as the temperature, providing a compromise between the transparency of the sample and the presence of a crystalline phase in it. At this temperature, only samples heated for 24 h became opaque.

### 3.3. FTIR Analysis

**Figure 5:**
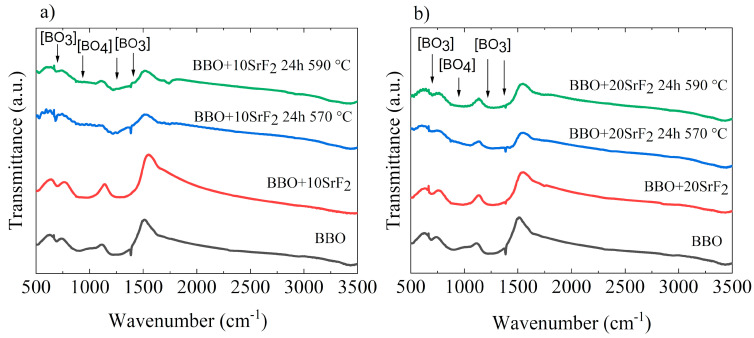
FTIR spectra of BBO+10SrF2 (**a**) and BBO+20SrF2 (**b**) as-prepared glasses and glasses after annealing at 570 °C and 590 °C. For comparison, the spectrum of as-prepared BBO glass was added to each figure.

Changes in the structure of the studied glasses occurring due to annealing were also visible in the FTIR spectra. The FTIR spectra obtained for BBO+10SrF_2_ and BBO+20SrF_2_ glasses and glass-ceramics are shown in Figure 5a,b, respectively. For comparison, the spectrum of as-prepared BBO glass was added to each figure. It is well known that the network of borate glasses is mainly constructed from [BO_3_] triangular structural units, which under the influence of the modifier can transform into [BO_4_] tetrahedral units. Such a change can also occur under the influence of Bi_2_O_3_ [12]. The broad absorption bands in the region of 680–720 cm^−1^ and 1100–1250 cm^−1^ are usually assigned to deformation and stretching vibrations of [BO_3_] groups [10,13,33]. In addition, the band at about 1200–1400 cm^−1^ can be attributed to the stretching vibration of [BO_3_]. On the other hand, the presence of [BO_4_] groups may be indicated by the band at 900–1100 cm^−1^ [13,33]. However, it should be noted that these bands may shift under the influence of Bi_2_O_3_ and overlap with the Bi_2_O_3_ peaks and shoulders [33]. Looking at the spectra presented in Figure 5, it can be concluded that the annealing did not significantly affect them. 

### 3.4. XPS Analysis

**Figure 6:**
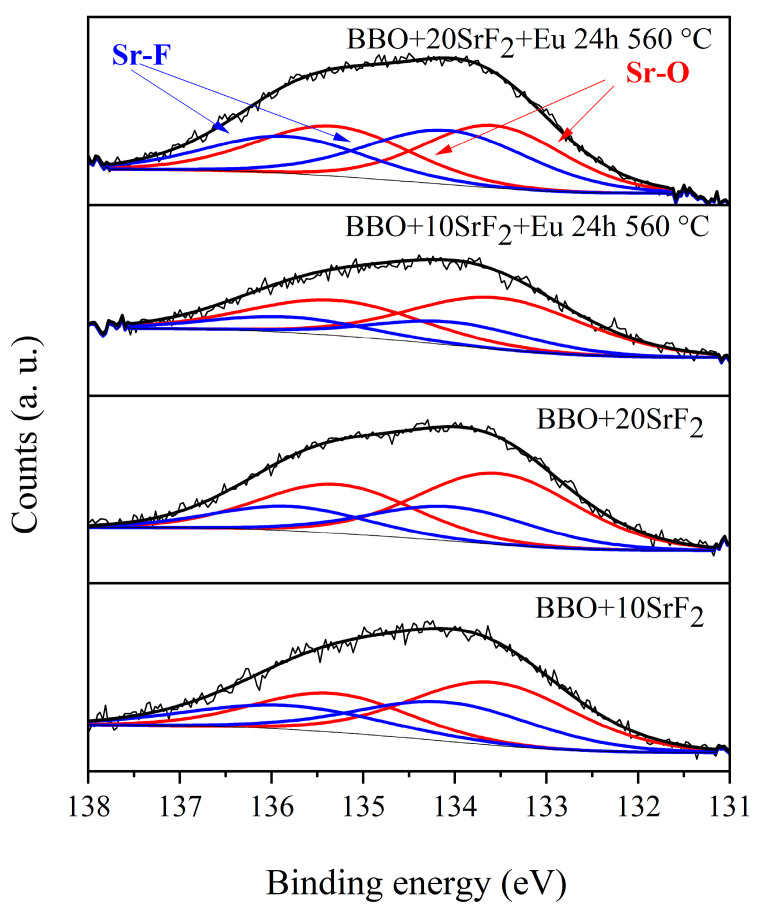
Sr 3D region of XPS spectra of BBO+10SrF_2_ and BBO+20SrF_2_ as-prepared glasses and glasses after annealing at 560 °C for 24 h.

XPS analysis was performed to provide additional information about the valence states of the elements of which the samples consisted. Particular emphasis was placed on the study of the valence states of Eu and Bi ions, as well as the chemical states of Sr that could indicate Sr chemical bonds with both fluorine and oxygen [24,34,35]. As research has shown, both Eu and Bi ions are in the 3+ valence state. Exemplary spectra of the Sr region of BBO+10SrF_2_ and BBO+20SrF_2_ glasses and glass ceramics after annealing at 560 °C for 24 h are shown in Figure 6. All spectra consisted of the Sr 3d spin-orbit doublet, but a detailed analysis of these peaks suggests the presence of more than one chemical state of Sr in all samples. Peaks at 133.5 and 135.5 eV could be assigned to the Sr 3d_5/2_ and Sr 3d_3/2_ in Sr-O [34,35], whereas peaks with energies equal to 134.0 and 136.0 eV could be attributed to Sr 3d_5/2_ and Sr 3d_3/2_ in Sr-F [34,35]. The contribution of Sr-O and Sr-F doublets in the glasses was 59% and 41% (BBO+10SrF_2_) and 67% and 33% (BBO+20SrF_2_), respectively. The contribution of Sr-O and Sr-F doublets after annealing was, respectively, 80% and 20% (BBO+10SrF_2_) and 52% and 48% (BBO+20SrF_2_). Therefore, as can be seen, annealing did not change the ratio of the doublets in the same way in both samples. The increase in the contribution of the Sr-F doublet, which in turn indicated an increase in the amount of the SrF_2_ crystal phase in the matrix, only took place in samples containing 20 mol % SrF_2_. However, as the analysis of the X-ray diffractograms showed, SrF_2_ as the only crystalline phase was also present only in the samples containing 20 mol % SrF_2_. Therefore, it can be concluded from both of these methods that the formation of SrF_2_ nanocrystals hindered the crystallization of the matrix.

### 3.5. Luminescence Analysis

**Figure 7:**
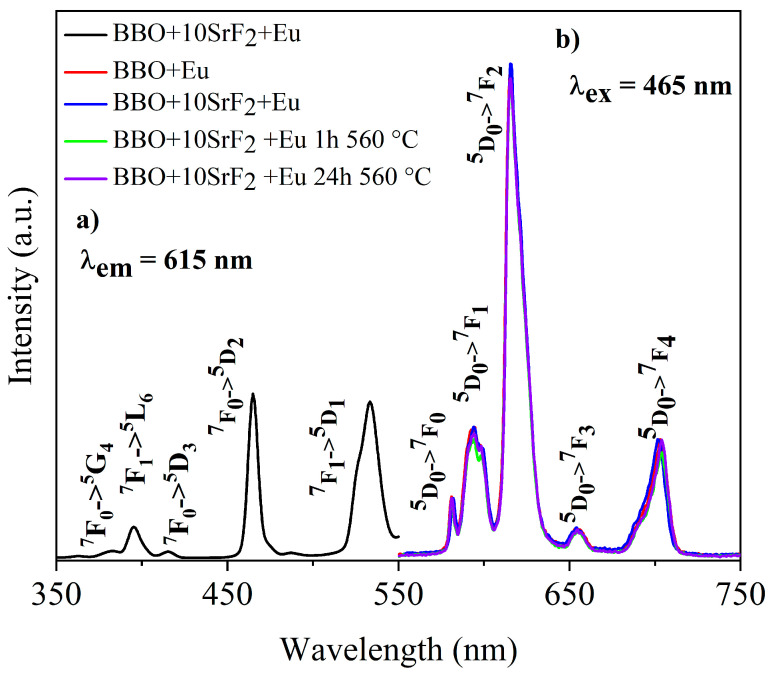
The excitation spectrum of BBO+10SrF_2_+Eu glass (**a**), and the emission spectra of BBO+Eu glass, and BBO+10SrF_2_+Eu glass and glass-ceramics after annealing at 560 °C for 1 h and 24 h (**b**).

**Figure 8:**
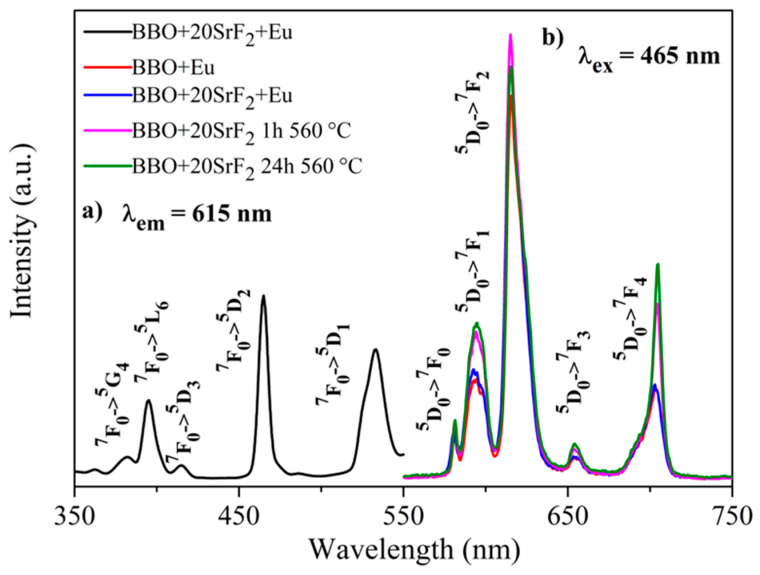
The excitation spectrum of BBO+20SrF_2_+Eu glass (**a**), and the emission spectra of BBO+Eu glass, and BBO+20SrF_2_+Eu glass and glass-ceramics after annealing at 560 °C for 1 h and 24 h (**b**).

Luminescence properties of Eu-doped BBO, BBO+10SrF_2_, and BBO+20SrF_2_ glasses and glass-ceramics were investigated to determine the influence of SrF_2_ on the luminescence intensity of Eu^3+^ ions. Figure 7a and Figure 8a show the excitation spectra of as-prepared BBO+10SrF_2_+Eu and BBO+20SrF_2_+Eu glasses monitored at the wavelength of λ_em_ = 615 nm, which corresponds to ^5^D_0_ → ^7^F_2_ transition of Eu^3+^ ions [3,36]. Several characteristics for 4f-4f transition peaks were visible in the spectrum. Excitation bands at 382 and 465 nm may have been assigned to ^7^F_0_ → ^5^G_4_, ^5^D_2_ transitions, whereas bands at 396, 415, and 533 nm originated from ^7^F_0_ → ^5^L_6_, ^5^D_3_, and ^5^D_1_ transitions, respectively [3,36]. A wavelength of 465 nm was selected for the observation of the emission spectra. This is the line with the highest intensity in the excitation spectrum. Emission spectra of BBO+10SrF_2_+Eu glass and glass-ceramics crystallized at 560 °C (Figure 7b) consisted of several bands corresponding to Eu^3+^ radiative transitions at 581 nm (^5^D_0_ → ^7^F_0_), 594 nm (^5^D_0_ → ^7^F_1_), 615 nm (^5^D_0_ → ^7^F_2_), 655 nm (^5^D_0_ → ^7^F_3_), and 703 nm (^5^D_0_ → ^7^F_4_) [3,36]. As can be seen, there were no significant differences between the intensity of the bands corresponding to the BBO+Eu glass and the glass with the addition of 10 mol % of SrF_2_. In addition, annealing of the BBO+10SrF_2_+Eu samples did not change the intensity of the spectral lines. However, if we look at the results concerning the structure of the annealed samples, the crystallization of the borane-bismuth matrix, apart from the SrF_2_ crystal phase, cannot be ruled out. Assuming that this is the case, the emitted radiation could undergo scattering on defects. Looking at the emission spectra corresponding to the BBO+20SrF_2_+Eu samples (Figure 8b), it can be noticed that the emission bands were at the same wavelengths as for the samples with 10 mol % of SrF_2_. In addition, it is evident that annealing affected the intensity of Eu^3+^ ions emission. It is worth noticing that the SrF_2_ crystalline phase was characterized by considerably lower phonon energy compared to the oxide materials. Therefore, if Eu^3+^ ions are located in the SrF_2_ nanocrystals, this leads to a decrease in multi-phonon relaxation probability, and consequently to an increase in emission efficiency. This was also the case with other strontium fluoride-containing glass ceramics [25,37].

### 3.6. Luminescence Decay and Judd–Ofelt Analysis

**Figure 9:**
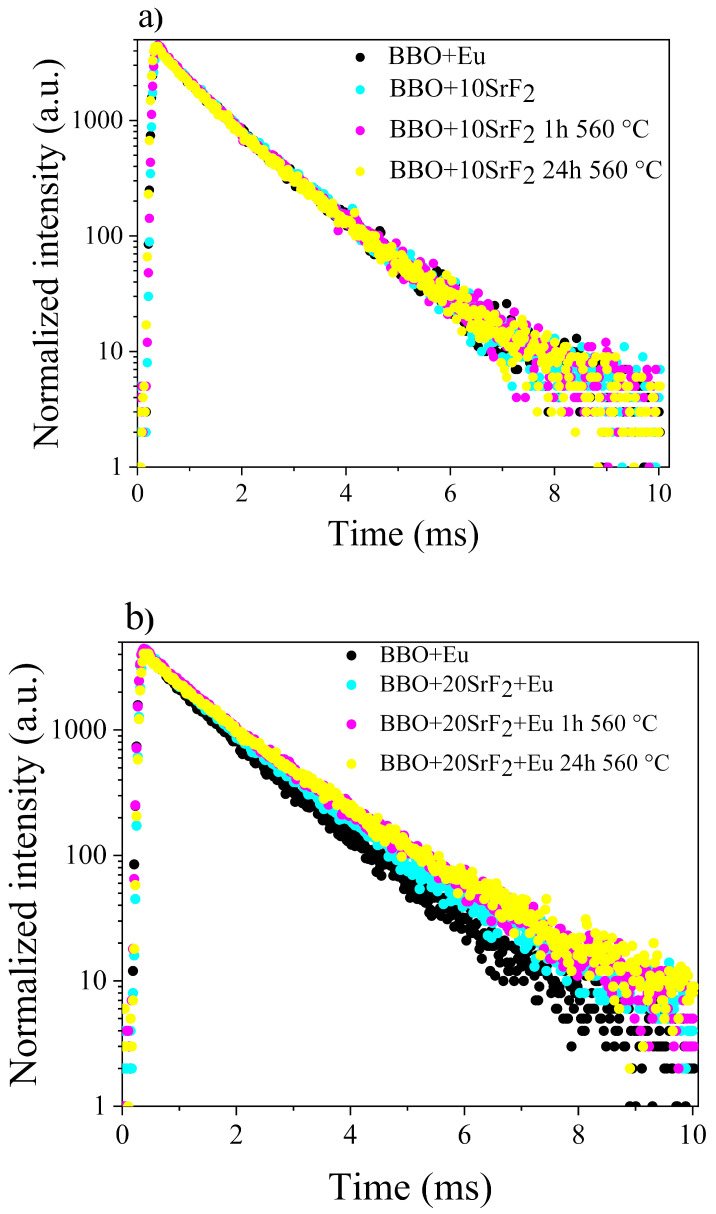
Luminescence decay curves of Eu-doped BBO and BBO+10SrF_2_ samples (**a**) Eu-doped BBO and BBO+20SrF_2_ samples (**b**).

Luminescence decay analysis was performed on as-prepared Eu doped BBO, BBO+10SrF_2_ and BBO+20SrF_2_ glasses and glass-ceramics. The decay curves were obtained by monitoring ^5^D_0_ →^7^F_2_ emission line (λ_em_ = 615 nm) upon excitation at λ_exc_ = 465 nm (^7^F_0_ →^5^D_2_ transition) and are shown in Figure 9a,b.

It was found that luminescence decays in both cases can be described as two-exponential decays according to the following equation [38]:

(2) $$I\left(t\right)=A_{1\;}\exp\left(\frac{-t}{\tau_{1}}\right)+A_{2}\exp\left(\frac{-t}{\tau_{2}}\right)$$

where τ_1_ and τ_2_ are long and short luminescence lifetime components contributing to the average lifetime 〈τ_avg_〉, and A_1_ and A_2_ are amplitudes of respective decay components. The lifetimes τ_1_ and τ_2_, amplitudes, and average lifetime calculated for the luminescence decay curves presented in Figure 9 are collected in Table 1.

This double-exponential nature of the decay curves indicates the presence of two different surroundings of Eu^3+^ ions. Long lifetime (τ_1_) is correlated with higher symmetry of the crystal field, whereas short lifetime (τ_2_) is associated with lower symmetry of the Eu^3+^ surroundings. Thus, in glasses containing SrF_2_ or PbF_2_ nanocrystals, a long lifetime is attributed to Eu^3+^ ions, which are located in the nanocrystals, and short lifetimes correspond to the ions incorporated into the amorphous matrix [24,25,37,39].

Concerning the tested samples, it can therefore be said that τ_1_ represents the lifetime of Eu^3+^ ions incorporated into the SrF_2_ nanocrystals, whereas τ_2_ corresponded to ions surrounded by the glass matrix. However, in the case of the studied samples, the situation seems to be more complicated. Two lifetimes were also observed in BBO+Eu glass, which did not contain strontium fluoride, and if to compare the results obtained for this glass with the values calculated for the BBO+10SrF_2_ sample, the lifetimes are not much different. This means that the tested glass may have contained nanocrystalline areas that were formed during the glass preparation process, which are not visible in diffraction studies. Especially in borate glasses with various dopants, single exponential decay curves are usually observed [40,41]. As can be seen, the longest τ_2_ lifetime was observed for the BBO+20SrF_2_ samples, especially those annealed for 24 h at 560 °C. It seems that in these samples the amount of Eu^3+^ ions incorporated into SrF_2_ nanocrystals increased. This result is in line with the results obtained with the XRD. They show that SrF_2_ as the only crystalline phase in the glass was present at 20 mol% of SrF_2_, whereas at 10 mol% the matrix crystallization also took place.

Changes in symmetry in the surroundings of the europium ions, as well as changes in the degree of covalence of bonds of these ions, can be observed based on Judd–Ofelt parameters. The Judd–Ofelt parameters Ω_2_ and Ω_4_ were calculated based on luminescence emission spectra using JOES application software and are presented in Table 1. Detailed information regarding software and calculations can be found in reference [42]. The Ω_6_ parameter was not determined in this study due to the unregistered emission band, located in the NIR range of wavelength, that corresponds to the ^5^D_0_ → ^7^F_6_ transition band. The Ω_2_ parameter is known to be structure sensitive and also depends on the covalence of Eu^3+^ bonds with the ligand [43,44,45,46]. It is determined from ^5^D_0_ → ^7^F_2_ hypersensitive transition. The high value of the Ω_2_ parameter (Ω_2_ > Ω_4_) suggests that Eu^3+^ ions occupied mostly low-symmetry sites. This corresponds to the situation where Eu^3+^ ions were mainly located in the glassy matrix. On the other hand, the Eu-O bond in the glasses was highly covalent, which was also reflected in the high Ω_2_ coefficients. In turn, the Ω_4_ parameter is related to the rigidity of glasses and is often attributed to the emergence of long-range effects related to crystal lattice [19]. Therefore, the processes of crystallization of glasses should consequently lead to an increase in this parameter. This behavior was observed, for example, in tellurite glass-ceramics containing SrF_2_ nanocrystals [25]. The parameters Ω_2_ and Ω_4_ calculated for the BBO+10SrF_2_ and BBO+20SrF_2_ glasses before and after annealing were different, but the change due to crystallization depended on the initial amount of SrF_2_. In the BBO+10SrF_2_ sample, both Ω_2_ and Ω_4_ were higher in the annealed samples. Nevertheless, it is a sample where it was difficult to say that SrF_2_ was the only crystalline phase in the glass matrix and it was, therefore, difficult to analyze the influence of the appearance of SrF_2_ nanocrystals on luminescence. On the other hand, in the case of the BBO+20SrF_2_ sample, after crystallization the parameter Ω_2_ was lower and Ω_4_ was higher than in as-prepared glass. However, in the BBO glass containing 20 mol% of SrF_2_, no additional crystallization of the matrix was observed after annealing; hence, it can be concluded that the observed change in the values of Ω_2_ and Ω_4_ parameters, as well as the increase in *τ*_1_ time, are because Eu^3+^ ions were located in SrF_2_ nanocrystals.

These results follow the luminescence intensity ratio R (asymmetry ratio), which in the case of Eu^3+^ ions can be calculated from the expression [45]:

(3) $$R=\frac{I\left({{}^{5}D}_{0}\to {{}^{7}F}_{2}\right)}{I\left({{}^{5}D}_{0}\to {{}^{7}F}_{1}\right)}$$

The transition ^5^D_0_ → ^7^F_1_ occurs via magnetic dipole and is independent of the host matrix, whereas ^5^D_0_ → ^7^F_2_ has a pure electric dipole moment origin and is hypersensitive to changes in the crystal field around Eu^3+^ ions. In other words, a more intense ^5^D_0_ → ^7^F_2_ transition indicates that the Eu^3+^ ions mainly occupy positions without an inversion center, whereas a more intense ^5^D_0_ → ^7^F_1_ transition shows that the Eu^3+^ ions are located at sites with higher symmetry. Therefore, with the change in site symmetry of Eu^3+^ ions, when they take positions with higher symmetry, the asymmetry ratio coefficient should decrease. The intensity ratios calculated for the as-prepared BBO+20SrF_2_ sample and the samples after annealing at 560 °C decreased with an increase in the annealing time (resulting in the growth of SrF_2_ nanocrystals). This means that some of the Eu^3+^ ions were located in the structure of nanocrystals. Unfortunately, as shown earlier, BBO glass initially containing 10 mol % of SrF_2_ may behave differently, which is most likely related to the not-completely-amorphous (after annealing) borate-bismuth matrix.

## 4. Conclusions

In summary, borate-bismuth glass-ceramics doped with Eu^3+^ ions, with SrF_2_ nanocrystals, were obtained. The structural modifications of parental glass, leading to SrF_2_ nanostructure crystallization, depend strongly on the initial amount of strontium fluoride. In the case of borate-bismuth glass, it is possible to obtain at least five stable crystalline phases of Bi_2_O_3_-B_2_O_3_ [11], and 10 mol % of SrF_2_ introduced into the glass is insufficient to block crystallization of the matrix. This has a strong influence on the luminescent properties. The expected increase in the intensity of emission bands was not observed in such glass ceramics, which was probably related to the scattering of the emitted radiation on various types of defects occurring during the annealing of the glasses. The increase in luminescence intensity was observed after annealing in samples containing 20 mol% SrF_2_. The luminescence lifetimes obtained for these glass-ceramics indicate that some of the Eu^3+^ ions were located in SrF_2_ nanocrystals. This was also confirmed by the analysis of the Judd–Ofelt parameters Ω_2_ and Ω_4_, and luminescence intensity ratio R. It can therefore be concluded that the glasses and glass-ceramics described in this work could be considered as potential candidates for LED phosphors.

## Figures and Tables

**Table 1:** Fitting parameters of the luminescence decays, calculated Judd–Ofelt parameters, and luminescence intensity ratios.

	BBO+Eu	BBO+10SrF_2_+E	BBO+20SrF_2_+Eu	BBO+10SrF_2_+Eu 1 h 560 °C	BBO+10SrF2+Eu 24 h 560 °C	BBO+20SrF2+Eu 1 h 560 °C	BBO+20SrF2+Eu 24 h 560 °C
A_1_	3913	4148	4267	3297	3431	3440	3338
A_2_	3237	3115	2951	3893	3387	3046	2692
τ_1_ (ms)	1.2	1.18	1.28	1.24	1.22	1.45	1.48
τ_2_ (ms)	0.56	0.5	0.52	0.58	0.56	0.66	0.65
*<τ_avg_>* (ms)	1	1	1.1	1	1	1.2	1.2
Ω_2_	5.64	5.15	5.54	6.06	5.86	4.48	5.11
Ω_4_	3.89	3.74	3.59	3.78	3.85	3.86	4.07
R/O	3.76	3.45	3.69	4.04	3.90	3.06	2.73

## Data Availability

The data presented in this study (FTIR and luminescence) are openly available at: https://mostwiedzy.pl/pl/open-research-data/luminescence-and-ftir-measurements-of-b2o3-bi2o3-srf2-glass-and-glass-ceramics-doped-with-eu3-ions,629025230630285-0 (accessed on 30 June 2020).
